# Identification and Characterization of Adipose Tissue-Derived Human Antibodies With “Anti-self” Specificity

**DOI:** 10.3389/fimmu.2020.00392

**Published:** 2020-02-28

**Authors:** Daniela Frasca, Alain Diaz, Maria Romero, Denisse Garcia, Diya Jayram, Seth Thaller, Maria del Carmen Piqueras, Sanjoy Bhattacharya, Bonnie B. Blomberg

**Affiliations:** ^1^Department of Microbiology and Immunology, University of Miami Miller School of Medicine, Miami, FL, United States; ^2^Sylvester Comprehensive Cancer Center, University of Miami Miller School of Medicine, Miami, FL, United States; ^3^Miami Integrative Metabolomics Research Center (MIMRC), University of Miami Miller School of Medicine, Miami, FL, United States; ^4^Division of Plastic and Reconstructive Surgery, Department of Surgery, University of Miami Miller School of Medicine, Miami, FL, United States

**Keywords:** adipose tissue, autoimmune antibodies, adipocytes, antigen presentation, B cells

## Abstract

We have previously shown that the human obese adipose tissue (AT) contributes to increased secretion of adipocyte-specific IgG antibodies in individuals with obesity. This occurs without any exogenous stimulation, because the ongoing process of cell death in the obese AT leads to the release of “self” antigens able to induce chronic stimulation of B cells. We have identified several mechanisms responsible for the release of “self” antigens, such as hypoxia, cell cytotoxicity, and DNA damage. In this paper, we confirm and extend our initial observation on a different cohort of individuals, and we show that also the plasma of these individuals is enriched in IgG antibodies with specificities for adipocyte-derived antigens. Adipocyte-specific IgG secreted in the obese AT are significantly correlated with those present in plasma. Using immunoprecipitation and mass spectrometry, we have identified these antigenic specificities. The antigens are almost exclusively intracellular or cell-associated, usually not recognized as “self” antigens, but they are released by cells dying in the AT. We also show for the first time that the adipocytes in the obese AT contribute to the secretion of IgG autoimmune antibodies and this seems to be due to their expression of the antigen-presenting molecules CD1d and, to a much lesser extent, MHC class II, as our mechanistic experiments performed in mice have shown. These results may lead to the development of novel therapeutic strategies to control autoimmunity.

## Introduction

Obesity is associated with reduced production of protective antibodies (1, 2) and increased production of autoimmune antibodies in mice (3) and humans (4). Obesity and related inflammation create an optimal environment for the development of autoimmune diseases, as it leads to a breakdown of mechanisms of anti-“self” tolerance, which also worsens disease progression. Results obtained in mice have shown that mice on high-fat diet, as compared to mice on normal-fat diet, have higher plasma levels of heat shock protein 60 (HSP60) which are associated with higher plasma levels of Hsp60-specific IgG2c (autoimmune) antibodies (3). Results obtained in humans have shown that the plasma of obese individuals with insulin resistance (IR) contains autoantibodies specific for intracellular proteins, ubiquitously expressed in tissues including pancreas, nervous tissues, muscle, or AT as well as immune cells (4), suggesting the release of “self” antigens under obesity conditions in insulin target tissues. The majority of “self” antigens are cell-associated proteins (Golgi and endoplasmic reticulum proteins, RNA polymerase, glutathione transferase, signaling proteins) with variable tissue expression.

To our knowledge, there are not published data showing secretion of antibodies with autoimmune specificities in the human obese AT, following local release of these “self” antigens. We have recently identified several mechanisms responsible for the release of “self” antigens in the human obese AT, such as hypoxia, NK cell cytotoxicity, and DNA damage, which induce cell death and further release of pro-inflammatory cytokines. We have also shown that adipocyte-specific IgG antibodies are secreted in the human obese AT and that AT-B cells express mRNA for activation-induced cytidine deaminase, the enzyme of class switch recombination and somatic hypermutation, as well as for the transcription factor T-bet and the membrane marker CD11c, both involved in the production of autoimmune IgG antibodies (5). In the present paper, we have characterized the “self” proteins that have stimulated the secretion of autoimmune antibodies in cultures of AT-derived immune cells. Moreover, we show that the adipocytes express antigen presentation molecules CD1d and to a lesser extent MHC class II, and therefore they are good antigen-presenting cells in the obese AT.

## Materials and Methods

### Subjects

Experiments were performed using discarded subcutaneous obese AT from females undergoing breast reduction surgery at the Division of Plastic and Reconstructive Surgery at the University of Miami Hospital (*n* = 20, 25–55 years), Body Mass Index (BMI, kg/m^2^) 31–39.

The individuals participating in the study did not have diseases and did not use medications known to alter immune responses. We excluded subjects with type-2 diabetes mellitus, congestive heart failure, cardiovascular disease, chronic renal failure, malignancies, renal or hepatic diseases, autoimmune diseases, infectious disease, trauma or surgery, pregnancy, or documented current substance and/or alcohol abuse.

Study participants provided written informed consent. Study was reviewed and approved by the University of Miami human subject research office with institutional review board IRB protocols #20070481 and #20160542, which reviews all human research conducted under the auspices of the University of Miami.

### Mice

Male C57BL/6 mice were purchased from the National Institutes of Aging and maintained in our AAALAC-certified facility. Mice were acclimated for at least 7 days before sacrifice. Mice with evidence of disease were not used in these studies. In the experiments herein we used 4 middle-age (12 months old) obese mice. All studies adhered to the principles of laboratory animal care guidelines and were IACUC approved (protocol #16-252).

### Isolation of Immune Cells From the AT

Epididymal mouse AT and subcutaneous human AT were harvested from obese mice and obese patients undergoing breast reduction surgeries, respectively, weighed and washed with 1X Hanks' balanced salt solution (HBSS), as we have previously described (5, 6). Briefly, the AT was washed, minced into small pieces, passed through a 70 μm filter and digested with collagenase type I (SIGMA C-9263) for 1 h in a 37°C water bath. Digested cells were passed through a 300 μm filter, centrifuged at 300 g in order to separate the floating adipocytes from the stromal vascular fraction (SVF) containing the immune cells. The adipocytes represent the “floating” fraction of the AT after collagenase digestion. Adipocyte preparations are not contaminated with immune cells. The SVF was then resuspended in ACK to lyse the red blood cells, washed, counted, and used for flow cytometry and *in vitro* experiments. The SVF is a mixture of mesenchymal, endothelial, and immune cells. The immune fraction of the SVF isolated from the AT of obese mice and humans contains M1 macrophages, Th1, Th17, Tγδ, IFN-γ-producing CD8+ T cells, B cells and type 1 Innate Lymphoid Cells, all of which contribute to the secretion of pro-inflammatory cytokines, chemokines, and adipokines, and to IR.

### SVF Cultures

After isolation, cells (2 × 10^6^/ml) were resuspended in complete medium (c-RPMI, RPMI 1640, supplemented with 10% FCS, 10 μg/ml Pen-Strep, 1 mM Sodium Pyruvate, and 2 × 10^−5^ M 2-ME and 2 mM L-glutamine). Cultures were left unstimulated for 10 days, then supernatants collected and measured by ELISA for the presence of adipocyte-specific IgG.

Cultures were also set up in the absence of macrophages (MΦ) which were removed by plastic adherence. Briefly, cells (2 × 10^6^/ml) were resuspended in 1X PBS, incubated for 3 h at 37°C in Petri dishes (Falcon 3003), then both non-adherent cells and MΦ were counted with an hemocytometer. MΦ depletion was confirmed by flow cytometry.

MΦ-depleted SVF were reconstituted with MΦ or with adipocytes. The ratio MΦ:immune cells (or adipocytes:immune cells) in SVF was equal to what we measured in *ex vivo* isolated SAT. Cultures of MΦ and adipocytes alone were used as negative controls. Cultures were left unstimulated for 10 days, then the supernatants were collected and adipocyte-specific IgG secretion was evaluated by ELISA.

Similarly, adipocytes and SVF were obtained from the epididymal fat pads of obese C57BL/6 mice, as previously described (7). Before MΦ and adipocytes were added back to the MΦ-depleted SVF cultures, they were treated for 1 h at 4°C with the following antibodies: anti-MHC class II (I-E kappa specific, 1:100 diluted, Abcam ab25681) or with anti-CD1d (1:100 diluted, Abcam ab119846).

### Preparation of Cytoplasmic Protein Extracts

To measure adipocyte-specific IgG in plasma and in the supernatants of SVF cultures, we prepared cytoplasmic protein extracts of the adipocytes. Briefly, cells were centrifuged in a 5415C Eppendorf microfuge (2,000 rpm, 5 min). The pellet was resuspended in 20 μl of a solution containing Hepes 10 mM, pH 7.9, KCl 10 mM, EDTA 1.0 mM, DTT 1 mM, MgCl_2_ 1.5 mM, PMSF 1 mM, 1 tablet of protease inhibitor cocktail (Boeringer Manheim) (per 20 ml), 1 mM Na_3_VO_4_ and Nonidet P-40 (0.1%), briefly vortexed and centrifuged (8,000 rpm, 5 min, 4°C). The supernatant containing the cytoplasmic extract was removed and stored at −80°C until use. Protein content was determined by Bradford (8). This is a colorimetric protein assay based on the binding of protein molecules to the Coomassie dye (ThermoFisher Scientific) which, under acidic conditions, results in a color change from brown to blue. Bovine serum albumin (BSA) at the concentration of 2 mg/ml is used as reference. Samples are read in a spectrophotometer set to a wavelength of 595 nm.

### ELISA

Adipocyte-specific IgG in plasma and in the supernatants of SVF cultures were measured by human Ig quantitative ELISA kits (Bethyl Labs E80-104). Briefly, cytoplasmic extracts from adipocytes at the concentration of 10 μg/ml in 1×PBS were used to coat ELISA plates. After 1 h at room temperature, plates were washed, blocked with 1×PBS containing 1% BSA (washing buffer) and then incubated for 30 min at 37°C. Then samples were added and incubated at room temperature for 3 h. Wells were washed thoroughly with washing buffer before receiving the detecting antibody goat anti-human IgG-Fc HRP-conjugated (1:5,000 diluted). After 1 h incubation at room temperature, wells were washed and given the substrate solution (TMB chromogen; Biosource SB01). Wells were incubated 15–20 min at room temperature to allow reactions to develop. Well contents were measured for absorbance at 405 nm.

### Mass Spectrometry (MS)

We used MS to identify “self” antigens that have induced the secretion of specific IgG antibodies in SVF cultures. The experimental procedure included the following steps: (1) enrichment of SVF culture supernatants in IgG antibodies (2) digestion of the IgG enriched supernatants with trypsin and generation of a mixture of tryptic peptides (3) acidification of the tryptic peptides to give them a positive charge (4) injection of the charged peptides into a High Performance Liquid Chromatography (HPLC) column, that allows separation of the peptides, and outflow to MS (5) fragmentation of the peaks and following computer matching of spectral patterns to determine amino acid sequences and identify proteins by correlating peptide-derived experimental MS spectra with theoretical spectra predicted from protein databases.

To enrich SVF culture supernatants in IgG antibodies, we used an immunoprecipitation kit (Dynabeads Protein G, ThermoFisher Scientific 100.07D), following manufacturer's instructions. All procedure was performed at room temperature. Briefly, Dynabeads Protein G were completely resuspended by vortexing, then 50 μl were transferred to a clean tube, placed on the column and supernatant separated on the magnet. Then the tube was removed from the magnet, IgG antibodies (10 μg) were added, gently mixed by pipetting and incubated with rotation 10 min. The tube was placed again on the magnet and supernatant removed. The sample containing the antigen (100 μl) was finally added and gently mixed by pipetting. After 10 min incubation with rotation, the tube was placed on the magnet, the Dynabeads/antigen/antibody complexes were thoroughly washed and the supernatant removed after each wash. Non-denaturing elution of target antigen took place in the tube after adding 20 μl of the elution buffer in rotation for 2 min (needed to dissociate the complex). The tube was placed on the magnet and the supernatant containing the eluted material was transferred to a clean tube.

After enrichment in IgG antibodies, samples were subjected to trypsinization, and then loaded onto an analytical column (75 μm i.d. × 15 cm, packed with Acclaim PepMap RSLC C18, 2 μm, 100 Å) connected to a precolumn (Acclaim PepMap 100 75 μm × 2 cm, nanoviper C18, 3 μm, 100 Å) for HPLC, attached to an Easy Nano LC 1000, all from ThermoFisher. This instrument was coupled to a QExactive, an Orbitrap mass spectrometer, also from ThermoFisher. Peptides were eluted following a gradient from 2 to 98% B for 1 h (solvents for chromatography were 0.1% formic acid (A) and acetonitrile (B) at 300 nL/min flow rate. The MS method was set to data-dependent acquisition (DDA) in positive mode, with an automatic gain control (AGC) of 1E6 for full MS, and 2E5 for MS^2^. The precursor tolerance was set to 10 ppm and 0.6 Da for fragment ions. HCD fragmentation was used, and the normalized collision energy (NCE) set up to 28 eV, with an isolation window of 1.3 m/z and a dynamic exclusion of 15 s. The bioinformatics analysis was performed using Proteome Discoverer v.2.1., from Thermo Fisher. Specific Uniprot human databases were downloaded and used for this analysis using SEQUEST HT as the search engine, with a relaxed False Discovery Rate (FDR) of 0.05, strict FDR of 0.01, and ΔCn of 0.05. The enzyme was set to trypsin in the analysis parameters, with a maximum of 2 missed cleavages, and a minimum length of 6 aminoacids.

### Immunofluorescence

Fresh tissue sections were obtained from the subcutaneous obese AT, they were placed in O.C.T. blocks (Tissue-Tek 4583) and stored at −80°C. Blocks were sectioned at 16 μm thickness and fixed in cold acetone for 10 min. For the staining, the tissue sections were fixed in acetone and washed twice with PBS. The tissue sections were then blocked for 1 h with 5% BSA at room temperature, and incubated with primary anti-CD1d antibody (abcam ab11076), or with primary anti-MHC class II antibody (abcam ab55152), both 1:100 diluted, overnight at 4°C. The next day, the slides were washed with PBS twice for 5 min and incubated with the following secondary antibodies: AF488-conjugated goat anti-mouse IgG (Biolegend 405319) for CD1d detection, and AF647-conjugated goat anti-mouse IgG (ThermoFisher InVitrogen A-21236) for MHC class II detection, both 1:100 diluted, 1 h at room temperature, or with the isotype controls [IgG1 (Biolegend 401402) and IgG2a (Biolegend 401502), respectively]. The slides where then washed 3 times, 3 min each. The cover slides were mounted with ProLong Gold Antifade Mountant and DAPI (4′,6-diamidino-2-phenylindole, ThermoFisher Scientific), which stains the nuclei of immune cells, to visualize the nuclei. Slides were imaged with a Keyence inverted microscope.

### Western Blotting (WB)

For the evaluation of specific proteins, cytoplasmic protein extracts from the adipocytes at equal protein concentration were denatured and then electro-transferred onto nitrocellulose membranes. Membranes were incubated with the following primary antibodies in PBS-Tween 20 containing 5% milk: mouse anti-human CD1d (1:500 diluted, BioLegend 350302), mouse anti-human MHC class II (1:500 diluted, abcam ab55152), mouse anti-human UBC9 (1:1,000 diluted, BD 617048). After overnight incubation with the primary antibodies, immunoblots were incubated with the following secondary antibodies for 3 h at room temperature: goat polyclonal anti-mouse IgG HRP-conjugated (1:50,000 diluted, Jackson ImmunoResearch Labs 115-035-003). Membranes were developed by enzyme chemiluminescence and exposed to CL-XPosure Film (Pierce). Films were scanned and analyzed using the AlphaImager Enhanced Resolution Gel Documentation & Analysis System (Alpha Innotech) and images were quantitated using the AlphaEaseFC 32-bit software.

### RNA Extraction and Quantitative (q)PCR

After isolation, adipocytes were resuspended in TRIzol (ThermoFisher Scientific) and sonicated, then RNA extracted for quantitative (q)PCR. Total RNA was isolated according to the manufacturer's protocol, eluted into 10 μl distilled water and stored at −80°C until use. Reactions were conducted in MicroAmp 96-well plates and run in the ABI 7300 machine. Calculations were made with ABI software. Briefly, we determined the cycle number at which transcripts reached a significant threshold (Ct) for each target gene and for GAPDH as control. A value of the target gene, relative to GAPDH, was calculated and expressed as ΔCt. Reagents and primers (Taqman) were from ThermoFisher Scientific.

### Statistical Anaslyses

Mean comparisons were performed by one way ANOVA or by Student's *t*-test (two-tailed), using GraphPad Prism version 7 software, which was used to construct all graphs.

## Results

### The Supernatants of SVF Cultures Are Enriched in Adipocyte-Specific IgG Antibodies and Are Correlated With Plasma Levels of IgG Antibodies With the Same Specificities

We have previously demonstrated that the supernatants of SVF cultures are enriched in adipocyte-specific IgG antibodies (5). This occurs without any exogenous stimulation, suggesting that the ongoing process of cell death in the AT leads to the release of “self” antigens able to induce chronic activation of B cells without the need of additional stimulation.

**Figure 1:**
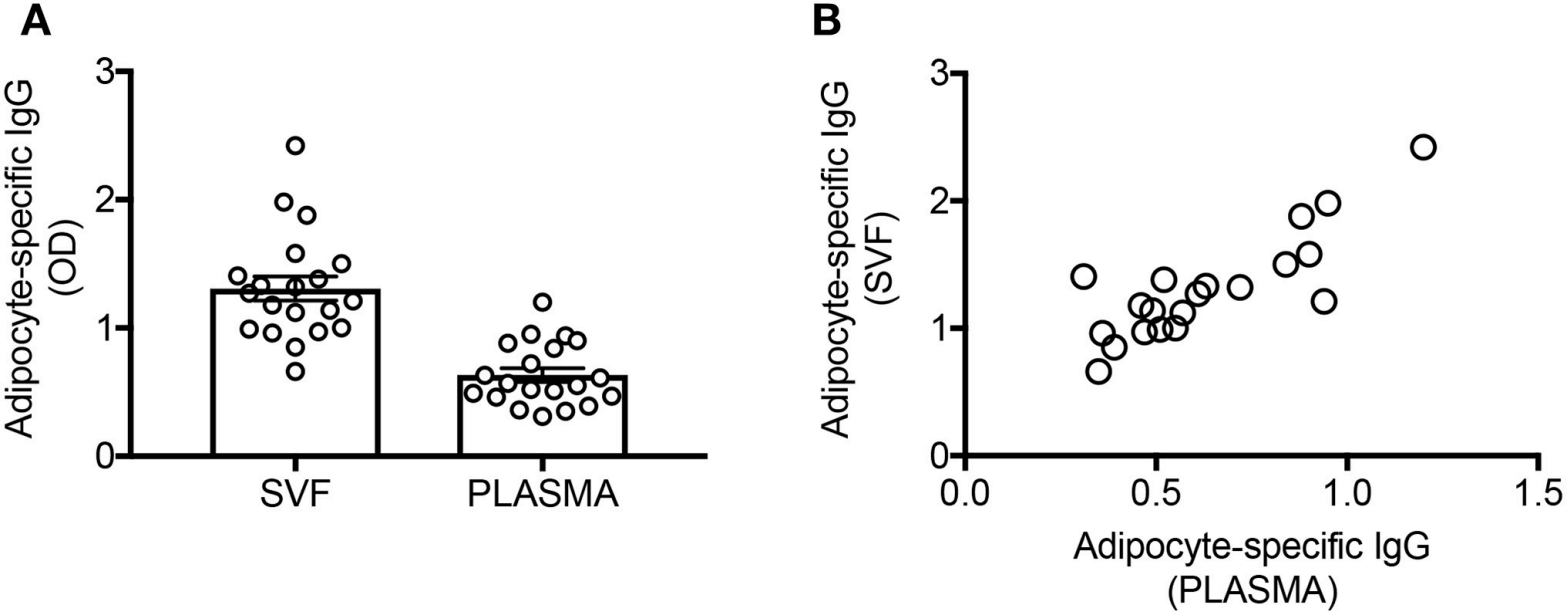
The supernatants of SVF cultures are enriched in adipocyte-specific IgG antibodies and are correlated with plasma levels of IgG antibodies with the same specificities. **(A)** Adipocyte-specific IgG antibodies were detected by ELISA in the supernatants of SVF cultures and in the plasma of obese individuals. **(B)** Pearson's *r* = 0.83, ****p < 0.0001.

Here we confirm (Figure 1A) and extend our initial observation on a different cohort of individuals, showing that the plasma of these individuals is also enriched in IgG antibodies with specificities for adipocyte-derived antigens. These specificities are only present in the plasma of obese but not lean adult individuals (9). Moreover, IgG in SVF cultures and in plasma from obese individuals are significantly correlated (Figure 1B).

### Identification of the Adipocyte-Specific IgG Antibodies Present in the Supernatants of SVF Cultures

**Figure 2:**
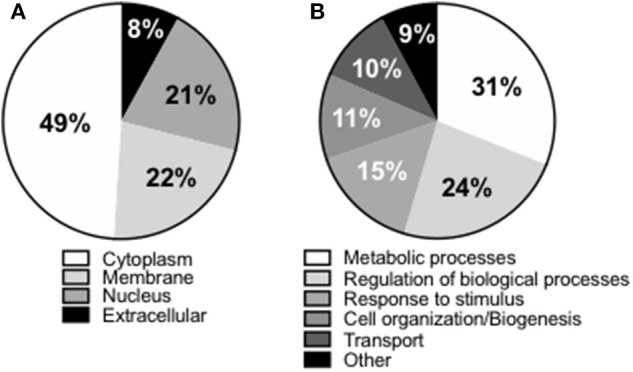
Compartmentalization of the “self” antigens. **(A)** SVF cultures (*n* = 9) were left unstimulated for 10 days, then supernatants collected, enriched in IgG using Protein G immunoprecipitation protocol, trypsin digested, and the antigens bound analyzed by MS. **(B)** Biological processes in which the “self” proteins are involved.

We next wanted to identify the adipocyte-derived antigens that stimulate IgG antibody secretion in SVF cultures. Briefly, SVF were left unstimulated for 10 days, then supernatants harvested and enriched in IgG by immunoprecipitation. Proteins in the supernatants of SVF cultures were trypsin digested and the tryptic peptides obtained were loaded on a HPLC coupled with MS. After fragmentation of the peaks, computer matching of spectral patterns was performed to determine amino acid sequences and a list of peptides was generated. We were able to identify several different antigenic specificities 16 of which were found in all the individuals tested (Table 1). These 16 antigens were almost exclusively intracellular or cell-associated (Figure 2A), usually not recognized as autoantigens, and they had various functions (Figure 2B). Antigens included signal transduction molecules, metabolic enzymes, hormones, cytoskeleton-associated proteins, DNA repair enzymes, histones. These results are the first to show that the supernatants of SVF cultures are enriched in IgG antibodies specific for adipocyte-derived antigens. The results generated by the MS experiment will help to create a protein array that will be used to screen plasma and SVF of individuals with obesity for the presence of adipocyte-derived IgG antibodies.

**Table 1:** Common “self” antigens inducing IgG secretion in the SVFs.

**Antigens**	**Cell localization**
Ubiquitin specific peptidase 4	Cytoplasm + plasma membrane
Tyrosine-protein kinase Fyn	Cytoplasm + plasma membrane
Sterol-C5 desaturase	Cytoplasm + plasma membrane
Fibronectin	Cytoplasm
Collagen alpha-1	Cytoplasm
Bruton Tyrosine-protein kinase	Cytoplasm
Glutathione S-transferase alpha-3	Cytoplasm
Fatty acid desaturase	Cytoplasm (ER + mitochondria)
Apolipoprotein B	Cytoplasm
Phosphatidylinositol 4,5-bisphosphate 3-kinase catalytic subunit beta isoform	Cytoplasm + nucleus
Enoyl-CoA hydratase domain-containing protein 3, mitochondrial	Cytoplasm + nucleus
Potassium voltage-gated channel subfamily A member 10	Cytoplasm + nucleus
Aspartoacylase	Cytoplasm + nucleus
Histone H2A	Nucleus
Histone-arginine methyltransferase CARM1	Nucleus
Heterogeneous nuclear ribonucleoproteins A2/B1	Nucleus

*SVF were left unstimulated for 10 days, then supernatants enriched in IgG by immunoprecipitation, trypsin digested and the antigens bound analyzed by MS*.

### Adipocyte Contribute to IgG Autoimmune Antibody Secretion in the AT

**Figure 3:**
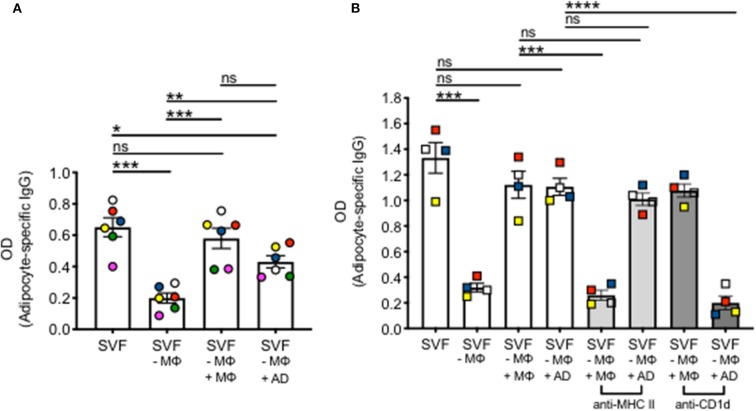
Adipocytes stimulate IgG autoimmune antibody secretion in the AT. **(A)** Human adipocytes and SVF were obtained from the same obese samples. MΦ were removed by plastic adherence after 3 h incubation at 37°C. MΦ were counted and added back to the SVF cultures from which they were removed. The ratio MΦ:immune cells (or adipocytes:immune cells) in SVF was equal to what we measured in *ex vivo* isolated AT. Different colors indicate samples from different individuals. **(B)** Mouse adipocytes and SVF were obtained from the epididymal fat pads of the same obese mice. MΦ and adipocytes were pretreated with anti-MHC class II (light gray columns) or with anti-CD1d (darker gray columns) before being added back to MΦ-depleted SVF cultures. AD, adipocytes. Different colors indicate different mice. Mean comparisons between groups were performed by one-way ANOVA. *p < 0.05, **p < 0.01, ***p < 0.001, ****p < 0.0001, ns, not significant.

The AT contains various immune cell types able to perform antigen presentation. These include MΦ, B cells and adipocytes. Adipocytes have only recently been shown to be antigen presenting cells, leading to T cell and invariant natural killer T (iNKT) cell activation, release of pro-inflammatory cytokines and establishment/maintenance of IR. The role of adipocytes on the secretion of autoimmune IgG in the AT, and especially in the human AT, has not been studied yet. We then investigated if adipocytes stimulate IgG antibody secretion *in vitro*. SVF cultures were left unstimulated for 10 days, then supernatants collected and adipocyte-specific IgG antibodies measured by ELISA. Secretion was compared to that in SVF cultures without MΦ, SVF cultures without MΦ to which MΦ were added back, SVF cultures without MΦ to which adipocytes were added back. Results in Figure 3A show adipocyte-specific IgG production in the supernatants of SVF cultures, as expected. Secretion is significantly reduced, but not completely abrogated, in MΦ-depleted SVF cultures. Adipocyte-specific IgG secretion is recovered not only when MΦ are added back, but also when adipocytes are added to the MΦ-depleted SVF cultures, strongly supporting the evidence that adipocytes have a role in IgG secretion in the AT. No adipocyte-specific IgGs were found in culture supernatants of MΦ alone or adipocytes alone (data not shown).

To evaluate if blocking MHC class II or CD1d had any effect on IgG secretion, we performed the following experiment in mice due to the difficulty to do mechanistic experiments with human AT samples (Figure 3B). Briefly, similar to the human experiments above, supernatants from unstimulated SVF cultures were evaluated by ELISA for the presence of adipocyte-specific IgG antibodies. Secretion was compared to that in SVF cultures without MΦ, SVF cultures without MΦ to which MΦ were added back, or those to which adipocytes were added back. Moreover, MΦ were pre-treated with an anti-MHC class II or with an anti-CD1d antibody, before being added back to MΦ-depleted SVF cultures. Adipocytes as well were pre-treated with the same antibodies before being added back to MΦ-depleted SVF cultures. Results show that pre-treatment of MΦ with anti-MHC class II but not anti-CD1d antibody decreased IgG secretion, whereas pre-treatment of adipocytes with anti-CD1d but not with anti-MHC class II decreased *in vitro* IgG secretion. These results suggest that antigen presentation by MΦ and adipocytes involves different antigen-presenting molecules, i.e., MHC class II and CD1d, respectively. We do not know at this point if the expression of MHC class II vs. CD1d is associated with the presentation of different antigens (protein antigens vs. lipid antigens) to different T cells [conventional CD4+ T cells (Tαβ) vs. Tγδ] by MΦ and adipocytes, respectively.

### Adipocytes Express the Antigen-Presenting Molecules CD1d and MHC Class II

**Figure 4:**
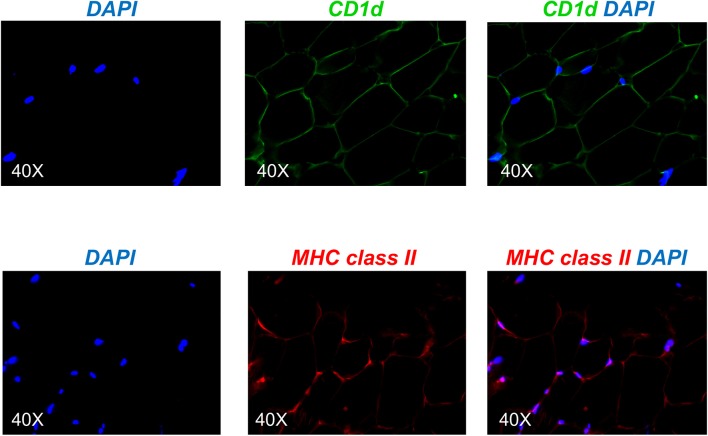
Detection of CD1d and MHC class II expression on the adipocytes by immunofluorescence. **(Top)** A section of AT was stained with anti-CD1d antibody followed by AF488-conjugated goat anti-mouse IgG. **(Bottom)** A section of AT was stained with anti-MHC class II antibody followed by AF647-conjugated goat anti-mouse IgG. Images show localization of CD1d or MHC class II on adipocytes, alone or together with DAPI. Results are representative of 8 independent experiments.

**Figure 5:**
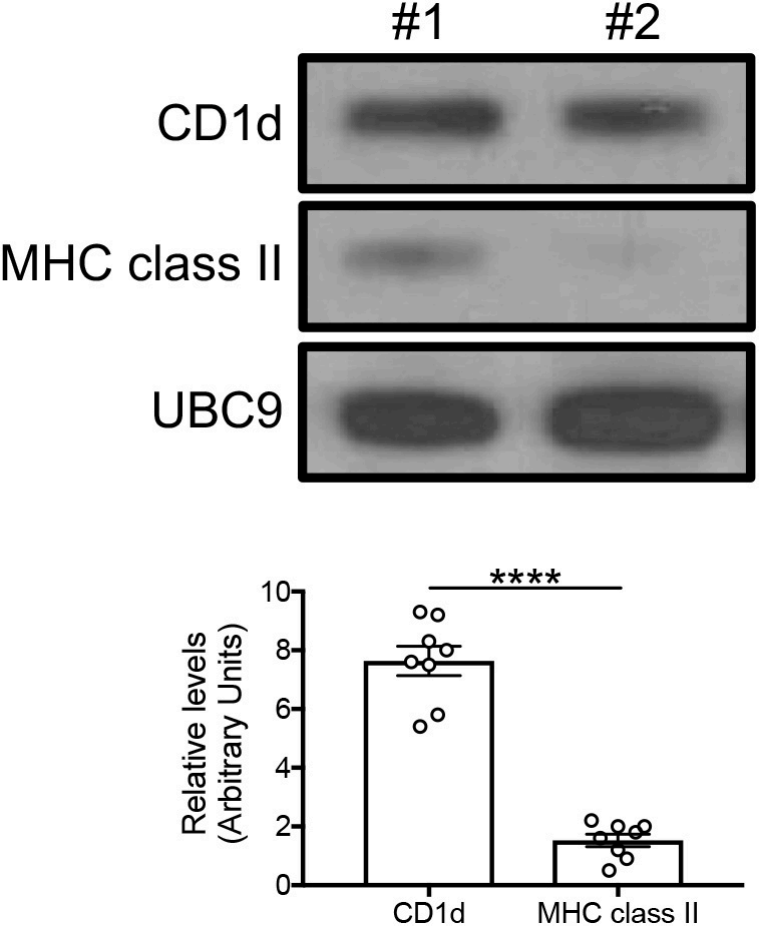
Detection of CD1d and MHC class II expression on the adipocytes by WB. Cytoplasmic protein lysates of adipocytes were prepared and run in WB to measure CD1d and MHC class II expression. **(Top)** A representative WB with 2 individuals is shown. **(Bottom)** Densitometric analyses (arbitrary units) of CD1d and MHC class II expression normalized to UBC9, are shown. ****p < 0.0001.

**Figure 6:**
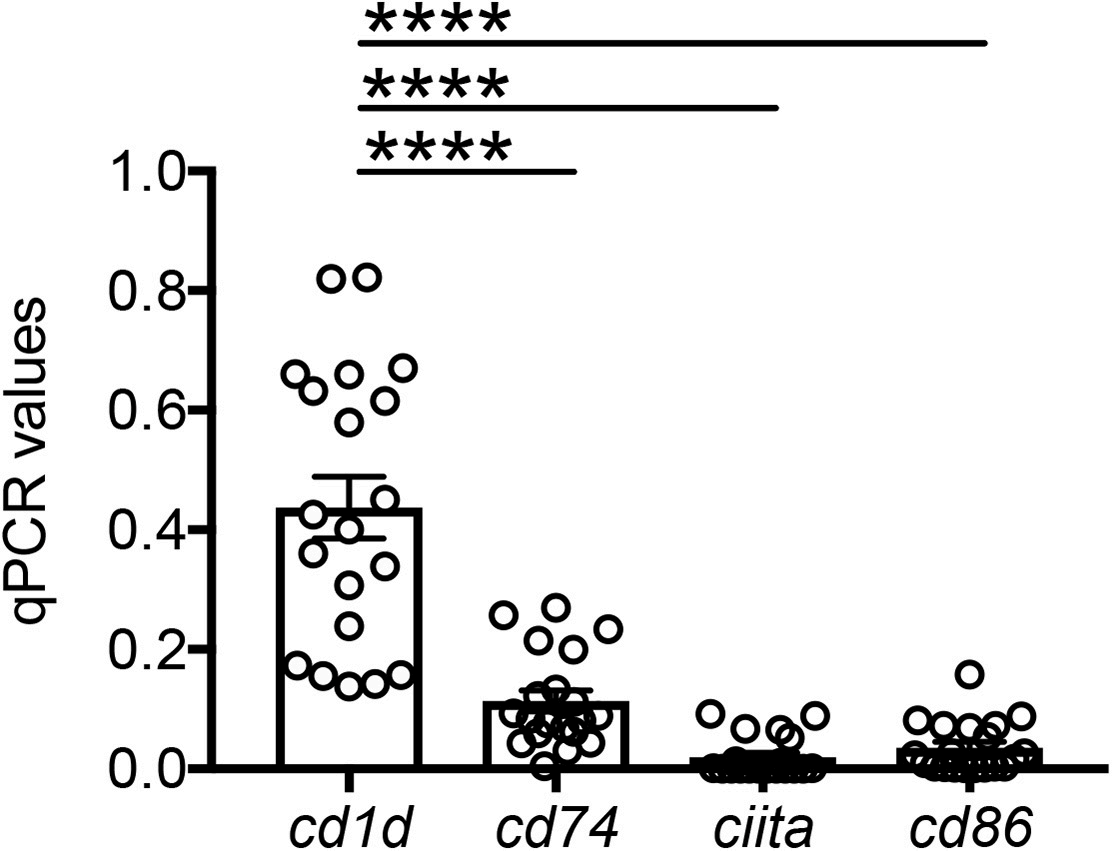
Detection of CD1d and MHC class II expression on the adipocytes by qPCR. Adipocytes were sonicated for cell disruption in the presence of TRIzol to separate the soluble fraction (used for RNA isolation) from lipids and cell debris. Results show qPCR values (ΔCt) of *cd1d, cd74, ciita, cd86*. Mean comparisons between groups were performed by one-way ANOVA. ****p < 0.0001.

Based on the mouse experiments presented in Figure 3B, we wanted to evaluate the expression of MHC class II vs. CD1d antigen-presenting molecules on human adipocytes. Both MHC class II (10–12) and CD1d (13, 14) have been shown to be expressed by mouse adipocytes. Nothing is known about the expression of these molecules on human adipocytes. We hypothesize that adipocytes may be primarily involved in the presentation of lipids and lipid antigens, a function associated with membrane expression of CD1d (13). Our immunofluorescence results in Figure 4 show for the first time that CD1d is expressed at higher levels than MHC class II in adipocytes from the human obese AT. Moreover, immune cells do not express CD1d (top) but MHC class II (bottom), as shown by the co-localization of red (MHC class II) and blue (DAPI) staining. These results were confirmed by WB (Figure 5) and qPCR (Figure 6) experiments. In qPCR experiments, we measured RNA expression of CD1d as compared with that of CD74 (MHC class II invariant chain peptide), CIITA (MHC class II transactivator) and CD86 (a major costimulatory molecule). These results altogether show that human adipocytes express predominantly, but not exclusively CD1d, strongly suggesting a role in the presentation of lipids and lipid antigens to T cells leading to the secretion of IgG antibodies with these specificities.

## Discussion

In this study, we show for the first time that the plasma of individuals with obesity is enriched in IgG with specificity for adipocyte-derived antigens. The serum of adult obese individuals with IR has been shown to contain autoantibodies specific for intracellular proteins, ubiquitously expressed in tissues including pancreas, nervous tissues, muscle or AT, as well as in immune cells (4), suggesting the release of “self” antigens under obesity conditions in insulin sensitive tissues. However, to date, there was no evidence that these autoantibodies were specific for AT-derived antigens. Not only the plasma of obese individuals, but also the supernatants of AT-derived immune cell cultures, are enriched in IgG specific for adipocyte-derived antigens. We have identified several antigenic specificities responsible for B cell activation and the secretion of autoimmune antibodies in the human obese AT. Using immunoprecipitation and MS, we were able to identify 16 antigenic specificities, that were found in all individuals evaluated. To our knowledge, there are not published data showing secretion of antibodies with autoimmune specificities in the human obese AT, following local release of these “self” antigens. We have previously identified several mechanisms responsible for the release of “self” antigens in the human obese AT, such as hypoxia, NK cell cytotoxicity, and DNA damage, which induce cell death and further release of pro-inflammatory cytokines. We have also shown that B cells in the obese AT express mRNA for the transcription factor T-bet, as well as the membrane marker CD11c, both involved in the production of autoimmune IgG antibodies (5).

Another important finding herein is that adipocytes contribute to the secretion of AT-specific IgG autoimmune antibodies *in vitro*, in both mice and humans. This is to our knowledge a novel finding. Traditionally, adipocytes function is to store excess energy. Adipocytes also secrete a large number of adipokines and pro-inflammatory mediators that stimulate AT inflammation. Very recently, adipocytes have been shown to work as antigen-presenting cells able to activate CD4+ T cells in the AT and drive local inflammatory responses (10). Experiments conducted in mice have clearly indicated that adipocytes express both CD1d (13, 14) and MHC class II (10, 11) antigen-presenting molecules. These results have suggested that adipocytes through antigen presentation activate pro-inflammatory cell subsets (Th1 CD4+ T cells, CD8+ T cells, and Tγδ cells) to release pro-inflammatory mediators, leading to the establishment and/or up-regulation of IR. In addition, adipocytes have been shown to be antigen-presenting cells for iNKT cells, which can be activated by endogenous lipids through CD1d, leading to IFN-γ release, up-regulation of AT inflammation and IR (13, 14). Adipocyte-activated iNKT cells may also increase the recruitment of immune cells, including MΦ, to the AT, further inducing up-regulation of AT inflammation and IR.

It is well-known that splenic murine iNKT cells provide help to B cells for lipid and protein-specific antibody responses, respectively (15), with the antibody response to lipids being characterized by cognate interaction leading to the generation of Germinal Centers (GCs) and affinity maturation of the antibodies dependent on iNKT cell-derived IL-21. Cognate help from iNKT cells, however, failed to drive classical T-dependent antibody responses. Conversely, the non-cognate help of iNKT cells to B cells has been shown in mice immunized with proteins and α-galactosylceramide (α-GC) (16). These mice produce higher levels of serum antibodies and show enhanced protection against influenza infection, as compared to mice immunized with proteins alone. Moreover, protein vaccination with α-GC, but not with conventional adjuvants, elicits IgG responses in mice lacking MHC class II molecules, demonstrating that iNKT cells can substitute for CD4+ T cell help to B cells. The direct help of NKT cells to B cells has also been observed in humans (17).

As opposed to splenic iNKT cells, there are no data on iNKT-B cell interaction in the AT. Moreover, it is not completely clear if the infiltration of iNKT in the AT is increased or decreased under obesity conditions. Mouse results have indicated decreased iNKT cell numbers in the AT of obese vs. lean mice, although these iNKT cells show higher levels of activation markers (13). Decreased iNKT cell numbers in the expanding AT under obesity conditions has been correlated with AT infiltration of pro-inflammatory MΦ (18). Increased iNKT cells in the mouse AT have also been shown (14). iNKT cells activated by CD1d-lipids rapidly secrete Th1, Th2, and Th17 cytokines. iNKT cells can also respond to “self” lipids presented by Dendritic Cells (DCs) in the presence of DC-derived cytokines. We can speculate that adipocyte-activated iNKT cells provide help to B cells, as iNKT cells recognize lipid-loaded CD1d expressed on B cells and directly provide helper signals which activate plasmablast expansion, GC formation, plasma cell differentiation, and IgG secretion.

Although we cannot exclude that adipocytes and B cells can also directly interact through CD1d, leading to B cell differentiation and antibody secretion, this possibility has not been evaluated yet. B cells, through CD1d expression, may form cognate interactions not only with iNKT cells but also with adipocytes. The human CD1 gene family is composed of five non-polymorphic genes (*cd1a, cd1b, cd1c, cd1d, cd1e*) (19), and it is expressed in all B cell subsets (20), including naïve and memory B cells, plasma cells, and B regulatory cells, in health and diseases, as reviewed in Chaudhry and Karadimitris (21). B cells have also been shown to be essential for iNKT cell expansion and activation in healthy individuals (22). Similar to MHC class II molecules, CD1d mediates the presentation of antigenic lipids on the surface of antigen-presenting cells after they have been loaded/processed in intracellular compartments; however, the mechanism by which this occurs remains uncharacterized. It would also be of interest to evaluate if MΦ can present lipids through CD1d and thereby activate iNKT cells and/or B cells. The effect of deleting CD1d in MΦ has not been addressed yet.

Adipocytes also express MHC class II antigen-presenting molecules in both mice (10, 11) and humans (10), as well as costimulatory signaling molecules such as CD80 and CD86 (10). Here we found that the expression of MHC class II is lower that that of CD1d on adipocytes. Studies in mice have shown that free-fatty acids promote adipocytes hypertrophy and increased expression of MHC class II (11). Leptin, an adipokine secreted primarily by the AT (23, 24), has also been shown to increase MHC class II expression on adipocytes (10). Leptin increases T cell proliferation and cytokine secretion, and expression of MHC class II and costimulatory molecule, in mice fed high-fat diet. It is not known if leptin is also able to increase the expression of CD1d. To our knowledge, no human data have shown so far on the up-regulation of antigen-presenting molecules by leptin.

We do not know at this point if the expression of CD1d vs. MHC class II by adipocytes is associated with the presentation of different antigenic specificities, for example lipid vs. non-lipid proteins, to different T cell subsets. The obese AT is heavily infiltrated with Tγδ cells which can preferentially recognize phospholipids (25), as well as glycolipids (26), presented by CD1d.

To summarize, in this study we show that the human obese AT is enriched in IgG antibodies specific for adipocyte-derived antigens, also found in plasma of individuals with obesity. The characterization of these antigens has shown that they are almost exclusively intracellular or cell-associated antigens, usually not recognized as “self” antigens, but they are released by cells dying in the AT. Moreover, adipocytes in the obese AT stimulate AT-specific IgG autoimmune antibody secretion, similar to professional antigen-presenting cells such as MΦ. The study of antibody responses against “self” antigens in the human AT is an exciting field of research that may help to design new therapeutic strategies of intervention, as well as new diagnostic tools, to control obesity and its associated complications.

## Data Availability Statement

All datasets generated for this study are included in the article/supplementary material.

## Ethics Statement

The studies involving human participants were reviewed and approved by University of Miami Human Subject Research Office with Institutional Review Board IRB protocols #20070481 and #20160542. The patients/participants provided their written informed consent to participate in this study. The animal study was reviewed and approved by IACUC protocol #16-252.

## Author Contributions

DF was involved in conceptualization, data curation and analysis, investigation, funding acquisition, writing, reviewing, and editing the manuscript. AD, MR, DG, DJ, MC, and SB performed the experiments, reviewed, and edited the manuscript. ST recruited surgery patients, collected adipose samples, reviewed, and edited the manuscript. BB was involved in conceptualization, reviewed, and edited the manuscript.

### Conflict of Interest

The authors declare that the research was conducted in the absence of any commercial or financial relationships that could be construed as a potential conflict of interest.
